# Multiplex Cytokine Analyses in Ear Canals of Dogs Suggest Involvement of IL-8 Chemokine in Atopic Otitis and Otodectic Mange—Preliminary Results

**DOI:** 10.3390/ani12050575

**Published:** 2022-02-25

**Authors:** Line-Alice Lecru, Daniel Combarros, Fabien Moog, Lukrecija Marinovic, Jevgenija Kondratjeva, Nicolas Amalric, Charline Pressanti, Marie Christine Cadiergues

**Affiliations:** 1 Department of Clinical Sciences, Université de Toulouse, ENVT, 31076 Toulouse, France; l.lecru13@hotmail.com (L.-A.L.); daniel.combarros@envt.fr (D.C.); fabien.moog@envt.fr (F.M.); lukre.marinovic@outlook.com (L.M.); jevgenija.kondratjeva@envt.fr (J.K.); charline.pressanti@envt.fr (C.P.); 2 INFINITy, Université de Toulouse, Inserm, CNRS, UPS, 31059 Toulouse, France; 3 QIMA Life Sciences, 31670 Labège, France; nicolas.amalric@qima.com

**Keywords:** dog, otitis, inflammation, atopic dermatitis, *Otodectes cynotis*, biomarkers, interleukin-8

## Abstract

**Simple Summary:**

Atopic dermatitis is a form of allergy of genetic origin accompanied by itching, during which the animal more easily develops allergies to environmental factors, usually pollen and dust mites. Among the symptoms, otitis is common. The mechanisms of this disease are still not fully understood. In this study, we aimed to demonstrate the detection ability of inflammatory markers (cytokines and chemokines) in the ear canals of atopic dogs suffering from otitis externa compared to healthy dogs and to dogs with parasitic otitis (inflammatory but not allergic otitis). Therefore, we non-invasively sampled the surface of the ear canals of atopic dogs and compared the amounts of certain cytokines and chemokines with those in similar samples taken from ears of healthy dogs and ears with spontaneous ear mite infestation. It appears that concentrations of IL-8 are significantly higher in atopic ears than in healthy ears. Nevertheless, this difference does not appear to be atopic-specific, since the amount of interleukin-8 (IL-8) also increased in ears infested with mites. Further investigations with a larger number of dogs are now required to confirm these results and possibly to find other biomarkers involved in the pathogenesis of canine atopic otitis.

**Abstract:**

Cutaneous cytokines and chemokines are involved in the pathogenesis of human and canine atopic dermatitis. The aim of the present study was to discriminate cytokine expression in the ear canals of atopic dogs with otitis, dogs with non-allergic inflammatory otitis (otodectic mange) and healthy non-atopic dogs. The ear canals of nine atopic dogs suffering from non-infected otitis externa (*n* = 14 ears), 10 healthy dogs suffering from otodectic mange (*n* = 20 ears) and 21 healthy controls (39 ears) were swabbed. The concentrations of a panel of 13 cytokines and chemokines on the aural surface were measured by multiplex analyses (Milliplex Canine Cytokine Panel). In addition, Canine Atopic Dermatitis Extent and Severity Index (CADESI)-04 and Otitis Index Score (OTIS3) scores were used to evaluate the overall status of the dogs. The concentration of IL-8 was significantly higher in the ears of atopic dogs and dogs with otodectic mange compared to those of healthy dogs. Significant increases in the levels of IL-10 were also overexpressed in atopic otitis but at lower rates. The concentrations of interleukin(IL)-8 were positively correlated with the OTIS3 hyperplasia score in atopic dogs. Taken together, these results suggest that IL-8 is overexpressed in atopic otitis and otodectic mange and that levels correlate with the otitis severity in atopic dogs.

## 1. Introduction

Canine atopic dermatitis (AD) is a genetically predisposed inflammatory and pruritic skin disease with characteristic clinical features, which is associated with immunoglobulin E(IgE) antibodies most often directed against environmental allergens [1,2].

The pathomechanism of canine AD is complex, involving a genetic background, a barrier defect, and an aberrant immune response to allergens and microbes. Affected dogs usually start showing pruritus between six months and three years of age. There is no sex predisposition, but several breeds are more prone to develop the disease (French Bulldog, English Bulldog, Chinese Shar-Pei, Wirehaired fox terrier, Golden retriever, Labrador retriever, Dalmatian, German shepherd dog, Boxer, Boston terrier, Lhasa Apso, Scottish terrier, Shih Tzu, and West Highland white terrier) [3]. Multiple genes seem involved with breed and geographical variations, making genetic screening tests difficult [4].

Clinical signs may be seasonal, non-seasonal, or non-seasonal with seasonal flares. Pruritus is the characteristic sign of AD. Predilection sites of affected skin are commonly the face, concave ear pinnae, axillae, groin, abdomen, perineum, ventral tail, and dorsal and ventral paws but lesion distribution can vary with the breed [3]. In addition, canine AD is the most common primary trigger for otitis externa in dogs [5,6,7]. Primary lesions are uncommon and consist of erythematous macules, patches, and small papules. Most lesions develop secondary to self-trauma and include alopecia, scaling, excoriations, lichenification, and hyperpigmentation. Secondary skin and ear infections with *Staphylococcus* and *Malassezia* spp. are common and often worsen the clinical signs. Chronic or recurrent otitis may be the only complaint in a small number of animals [3,6]. Dogs, irrespective of any atopic condition, can develop non-allergic ear infections, including otoacariasis or otodectic mange, which is by far the most common. The etiological agent of otoacariasis is *Otodectes cynotis*, an obligate parasitic mite that multiplies in the vertical and horizontal ear canals of dogs and cats. Dogs infested with *O. cynotis* most commonly develop otitis externa characterised by aural erythema and variable amounts of dark brown material [8].

Atopic dermatitis has been described for decades as a Th2 cell response, but recent evidence suggests a balance between lymphocyte phenotypes under the influence of a variety of cytokines, chemokines, and non-cytokine factors [9].

Inflammatory cytokines and chemokines have been identified in human and canine atopic skin linked to acute Th2 dominant response (Interleukin (IL)-4, IL-5, IL-13, IL-31, IL-33, thymic stromal lymphopoietin (TSLP), thymus and activation-regulated chemokine (TARC), granulocyte-macrophage colony stimulating factor (GM-CSF), monocyte chemotactic protein-1 (MCP-1)) in biopsy samples after epicutaneous application of allergens [10], in acute and chronic skin lesions [11] and in sera [12]. The acute episode precedes a Th1-dominated phase in chronic stages IL-2, interferon-gamma (IFN-γ), IFN-γ-inducible protein 10 (IP-10) [9,10,11,12,13,14,15]. Cytokine and chemokine expression in the external ear canal of atopic dogs has not previously been investigated.

The aim of this study was to measure concentrations of a panel of cytokines on the epithelium surface of the external ear canal sampled non-invasively in atopic dogs and to compare the results with those obtained in heathy dogs with normal ear canals (negative controls) and in dogs suffering from otodectic mange, a non-allergic inflammatory otitis externa (positive controls), all sampled in a similar way.

## 2. Materials and Methods

### 2.1. Animals

Client-owned dogs, over one year of age, irrespective of breed, sex, or weight were enrolled in three groups defined by the history and the clinical presentation. Both owner written consent and approval from the University Ethical Committee (SSA_2020_009) were obtained prior to beginning the study.

Group 1 comprised healthy dogs with no history of otitis or skin and general diseases. As the study was conducted in two steps, group 1 was divided in two sub-groups, group 1A, which was selected at the same time as group 2, and group 1B, which was selected at the same time as the group 3 dogs.

Group 2 included atopic dogs, otherwise in good overall health, apart from a documented history of chronic, non-seasonal canine AD with an active non-infected and untreated one-sided or bilateral otitis externa. Canine AD diagnosis was based on published criteria [16], compatible history, and clinical signs and exclusion of other pruritic conditions (flea allergy dermatitis, sarcoptic mange, and bacterial and/or fungal dermatitis). Cutaneous adverse food reaction was excluded based on a negative response to a minimum eight-week novel protein or hydrolysed elimination diet.

Dogs with evidence of poor body condition, suppurative otitis, and infectious erythemato-ceruminous otitis were excluded. Patient diet was at the discretion of the owner (including essential fatty acid supplementation) but had to be strictly unchanged over the two months prior to inclusion. For ethical reasons and because the study focused on the ear canals which were to be inflamed, canine AD background immunomodulatory treatment (ciclosporin, oclacitinib, and glucocorticoids) was allowed, provided it had remained unchanged during the three months preceding inclusion. For the same reasons, dogs receiving concurrent allergen-specific immunotherapy could be enrolled provided the dog had been receiving this therapy for a minimum of one year preceding inclusion. Dogs must not have received antimicrobial therapy or aural treatment over the four or the three weeks preceding enrolment, respectively.

Group 3 comprised dogs diagnosed with otodectic mange, but otherwise free of skin and general diseases. The diagnosis of otoacariasis was confirmed by detecting the presence of live mites either by video-otoscopy or on cerumen examination under a microscope [17].

### 2.2. Clinical Evaluation

Baseline data (signalment, physical examination) were collected at enrolment. Owners were asked to assess the severity of the aural pruritus on a visual analogue scale (VAS) score, consisting of a 10-cm horizontal line. Owners were instructed to place a mark on the VAS line at the location that best represented their dog’s aural itch. The distance in centimetres from the bottom of the line was measured and recorded for each case.

Ears were examined with a video-otoscope (Dailyscope, Optomed, Les Ulis, France) and a 0–3 Otitis Index Score (OTIS3) was used to evaluate the severity of the otitis [18]. Erythema, hyperplasia, erosion/ulceration, and exudate of both the vertical and horizontal ear canals were assessed on a 0–3 scale to give a total score of 0–12. In group 1, only ears with OTIS3 score ≤ 1, with both hyperplasia and ulceration = 0, were enrolled. In group 2, only ears with OTIS3 score ≥ 3, with ulceration = 0 were enrolled. In group 3, ears with parasitic mite *O. cynotis* visualised by video-otoscopy and/or identified by microscopy examination, were enrolled in the study. OTIS3 score was ≥1 with ulceration = 0.

The Canine Atopic Dermatitis Extent and Severity Index (CADESI)-04 was used to assess the severity of skin lesions. The severity of erythema, lichenification, and alopecia/excoriation was assessed at 20 body sites using a 0–3 scale [19].

### 2.3. Cytological Evaluation

Cytology was performed by collecting exudates with a standard dry non-sterile cotton swab from the junction of the vertical and horizontal ear canals and making a smear on a glass slide. Slides were dried and stained using the RAL 555 kit^®^ (RAL Diagnostics, Martillac, France) stain with dips for 15 s each time, according to the manufacturer’s recommendation. The slides were rinsed with tap water and dried. Ten fields were inspected on each slide under ×1000 magnification. Cytology scores (CYTO) from 0 to 4 were assigned to the presence of micro-organisms (cocci, rod-shaped bacteria, yeasts): 0, no microbe; 1, occasional microbes in a few high-power fields (HPFs); 2, small number of microbes (<5) on all HPFs; 3, moderate numbers of microbes on all HPFs; 4, high number of microbes on all HPFs [20,21].

In groups 1 and 2, only ears with a score below or equal to 1 were included (CYTO ≤ 1). In group 3, ears with a score below or equal to 2 (CYTO ≤ 2) were enrolled, because of the related microbial proliferation that commonly accompanies excessive cerumen production.

### 2.4. Sample Collection

In a preliminary step, exudate was removed and the ear canal was cleaned by rubbing gently with a dry sterile swab (MW112 Dryswab, Medical Wire & Equipment, Corsham, UK), which was then discarded. Subsequently, two swabs soaked with an aqueous non-ionic surfactant solution (QIMA Life Sciences proprietary method) were introduced and rubbed over the whole surface of the canal for 5 s. The swab heads were cut from the handle, placed in dry Eppendorf tubes and frozen at −20 °C until analysis.

### 2.5. Biochemical Prospection

Luminex^®^ technology was performed using a large predefined kit (Milliplex canine cytokine panel, #CCYTMG-90K-PX13, Merck, Darmstadt, Germany) for 13 parameters: IL-2, IL-6, IL-7, IL-8, IL-10, IL-15, IL-18, tumour necrosis factor (TNF)-α, IFN-γ, granulocyte-macrophage colony-stimulating factor (GM-CSF), IP-10, monocyte chemotactic protein-1 (MCP-1), and keratinocyte-derived chemokine (KC)-like. The method was applied according to the manufacturer’s instructions. All samples were analysed in duplicate. The minimum and maximum detection limits were 12 and 50,000 pg/mL, respectively, for all analytes except IFNγ (2 and 10,000 pg/mL, respectively). The kit contained internal controls corresponding to one low and one high concentration for each analyte. Additionally, the biomarker of interest was validated using an enzyme-linked immunosorbent assay (ELISA) (RayBio^®^ Canine IL-8 (CXCL8) ELISA, RayBiotech Life, Peachtree Corners, GA, USA) (groups 1A and 2).

### 2.6. Statistical Analysis

Concentrations were analysed using the Kolmogorov–Smirnov test. Correlations of variables were evaluated using a Spearman rank correlation for non-parametric data and linear regression analyses were applied. The results were analysed using XLStat 2020.4.1 and Microsoft Excel version 2016. A *p*-value < 0.05 was considered significant.

## 3. Results

### 3.1. Animals 

The negative control group (group 1) was composed of 21 dogs of various breeds, 10 males and 11 females aged 2–13 years (median = 5.5 years). Group 1A comprised only 19 ears, as one sample could not be analysed. All the dogs but one had a negative VAS score. The CADESI-04 score ranged between 0 and 11 (median = 4). The OTIS3 score ranged between 0 and 1 (median = 0) (Table 1).

Atopic dogs (group 2) consisted of one female and seven male dogs of various breeds, aged 1–15 years (median = 4.3 years). VAS score ranged between 0 and 5.45 (median = 2.9). CADESI score ranged between 6 and 45 (median = 24). One dog had a low score (CADESI-04 = 6) due to stabilisation of the disease in recent months due to immunomodulatory treatments which had been discontinued three months prior to inclusion. The dog, nevertheless, presented with a moderate to severe inflammatory otitis (left ear OTIS3 = 4, right ear OTIS3 = 6). In total, 14 ears were included. OTIS3 score ranged between 3 and 6 (median = 4). Three dogs were receiving immunomodulatory treatments glucocorticoids (2) and ciclosporin (1) at the time of enrolment. Nevertheless, their AD was not well-controlled (CADESI scores of 17, 35, and 45) and they had recently developed an aural flare (OTIS3 scores of 3, 5, and 3, respectively). None were on allergen-specific immunotherapy.

Group 3 consisted of four females and eight male dogs, aged 2–10 years (median = 5). The VAS score ranged from 0 to 2 (median = 0). The CADESI score ranged from 2 to 11 (median = 5). A total of 20 ears were included in this group and the OTIS3 ranged from 1 to 2 (median = 1).

### 3.2. Cytological Evaluation

In group 1A and 1B, respectively, four (21%) and six (30%) ears had a negative cytological score (CYTO = 0) and 15 (79%) and 14 (70%) ears had occasional bacteria or yeasts in a few fields (CYTO = 1).

In group 2, one ear (7%) had a negative score (CYTO = 0) and 13 ears (93%) had a score of 1 (CYTO = 1). In group 3, all ears had a positive cytological score; nine ears (45%) had a score of 1 (CYTO = 1) and 11 ears (55%) had a score of 2 (CYTO = 2).

### 3.3. Cytokine Concentrations in Ear Canals

**Figure 1:**
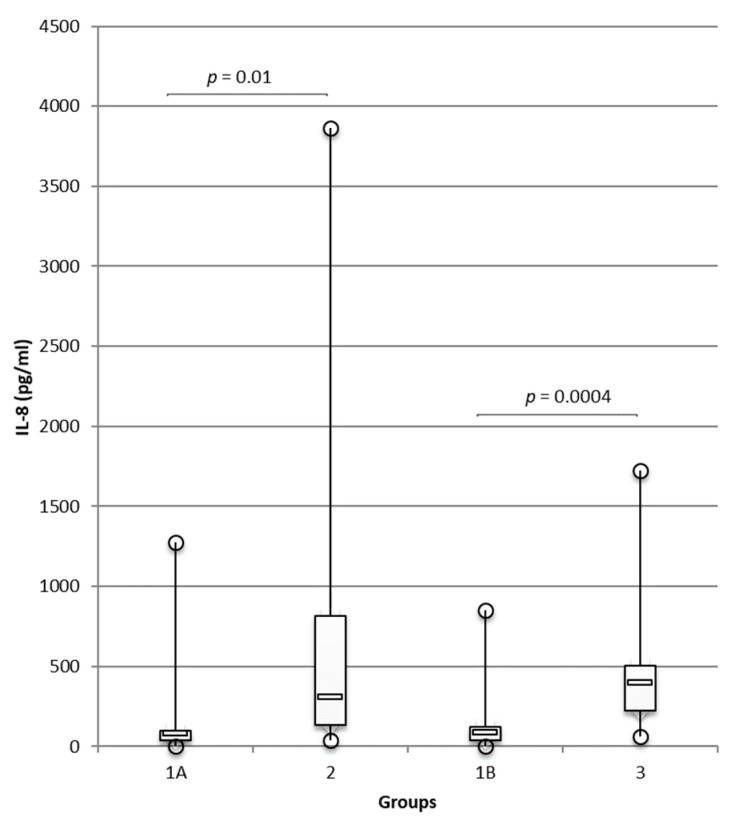
Increased IL-8 concentrations in ear canals of dogs suffering from atopic dermatitis/otitis and otodectic mange compared with healthy controls. Concentrations of IL-8 were measured with Luminex^®^ technology in the ear canals of healthy dogs, group 1A (*n* = 19 ears), and group 1B (*n* = 20 ears), and were compared, respectively, with those in atopic dogs with otitis, group 2 (*n* = 14 ears), and dogs with otodectic mange, group 3 (*n* = 20 ears), using the multiplex cytokine panel. Data are presented as box and whiskers (median, interquartile range, and min–max values). For greater readability, the maximum IL-8 concentration in group 2 was removed from the data analysis; the authors have assumed that this removal does not negatively affect their objective, and differences in IL-8 concentrations between the healthy group and the atopic group were significant with or without this value (*p* = 0.002 and *p* = 0.010, respectively).

**Figure A1:**
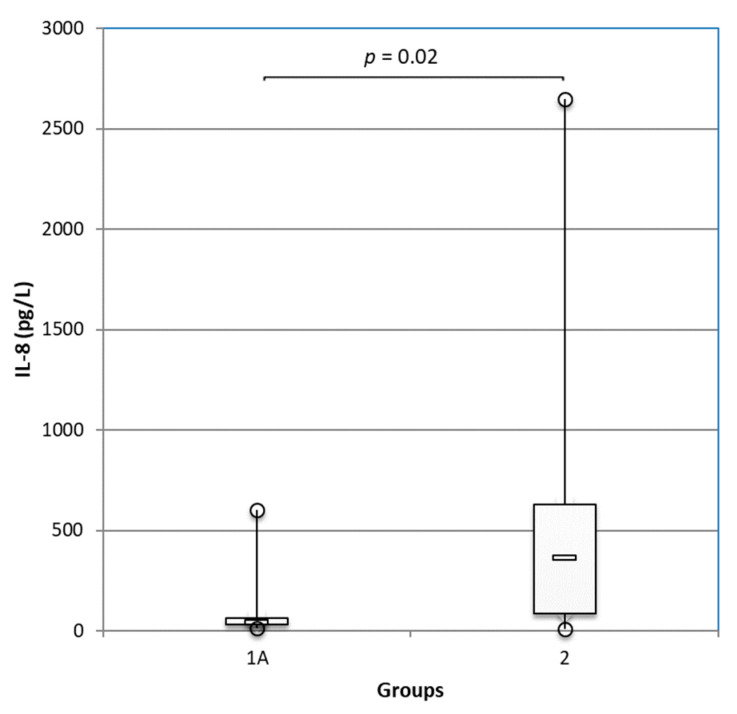
Increased IL-8 in the ear canals of dogs suffering from atopic dermatitis/otitis and otodectic mange compared with concentrations in healthy controls. Concentrations of IL-8 were measured with an ELISA test in the ear canals of healthy dogs, group 1A (*n* = 19 ears), and group 1B (*n* = 20 ears), and were compared, respectively, with atopic dogs with otitis, group 2 (*n* = 14 ears), and dogs with otodectic mange, group 3 (*n* = 20 ears), using the multiplex cytokine panel. Data are presented as box and whiskers (median, interquartile range, and min–max values).

Cytokine concentrations in the ear canals of atopic dogs (group 2) and dogs with otodectic mange (group 3) were analysed by the multiplex panel and compared with those in the ears of healthy dogs (groups 1A and 1B, respectively). In all groups, the samples analysed displayed detectable concentrations of IL-8, IL-10, IFN-γ, KC-like, and MCP-1, while IL-2, IL-6, IL-7, IL-15, IL-18, TNF-α, GM-CSF, and IP-10 were undetectable. Concentrations of IL-8 were significantly higher in group 2 than in group 1A (*p* = 0.01; Figure 1) and this result was confirmed by an ELISA (*p* = 0.01; Appendix A, Figure A1). Concentrations of IL-8 were high with a mean concentration of 733 pg/mL in group 2.

IL-8 was also significantly overexpressed in group 3 compared with healthy controls in group 1B (*p* = 0.0004; Figure 1) and like in atopic dogs, the levels were high (mean = 440 pg/mL in group 3), but no significant differences were observed between groups 2 and 3.

**Figure 2:**
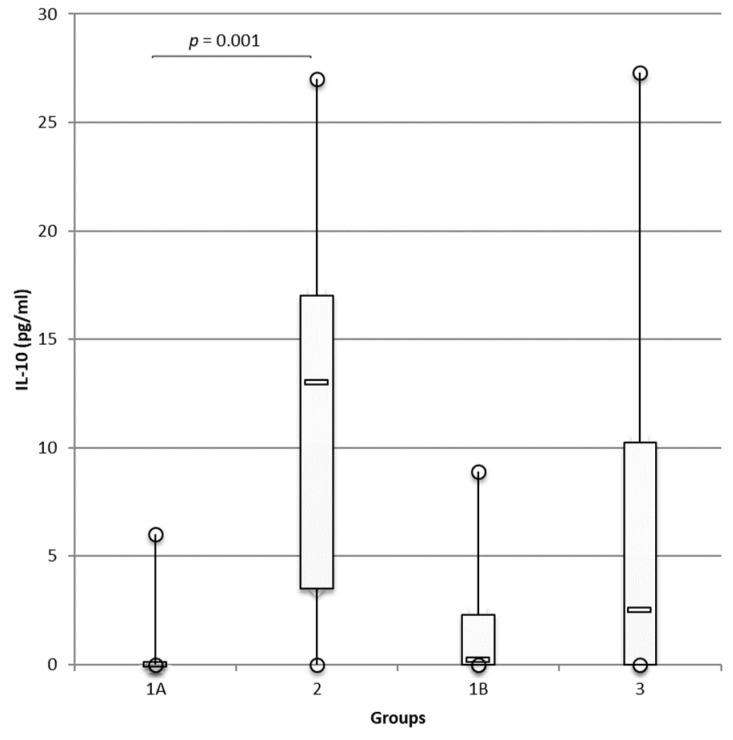
Higher IL-10 concentrations in ear canals of dogs suffering from atopic dermatitis/otitis and otodectic mange compared with those in healthy controls. Luminex^®^ technology was used to measure concentrations of IL-10 in the ear canals of healthy dogs, group 1A (*n* = 19 ears), and group 1B (*n* = 20 ears), and the concentrations were compared, respectively, with atopic dogs with otitis, group 2 (*n* = 14 ears), and dogs with otodectic mange, group 3 (*n* = 20 ears) using the multiplex cytokine panel. Data are presented as box and whiskers (median, interquartile range, and min–max values).

Similarly, IL-10 concentrations were significantly higher in group 2 than in group 1A (*p* = 0.001; Figure 2). IL-10 concentrations were also higher in group 3 than in group 1B control dogs, but the differences were not significant.

Concentrations of IL-10 were much lower than the concentrations of IL-8 with mean concentrations of IL-10 = 11.6 pg/mL and 5.9 pg/mL in groups 2 and 3, respectively.

**Figure 3:**
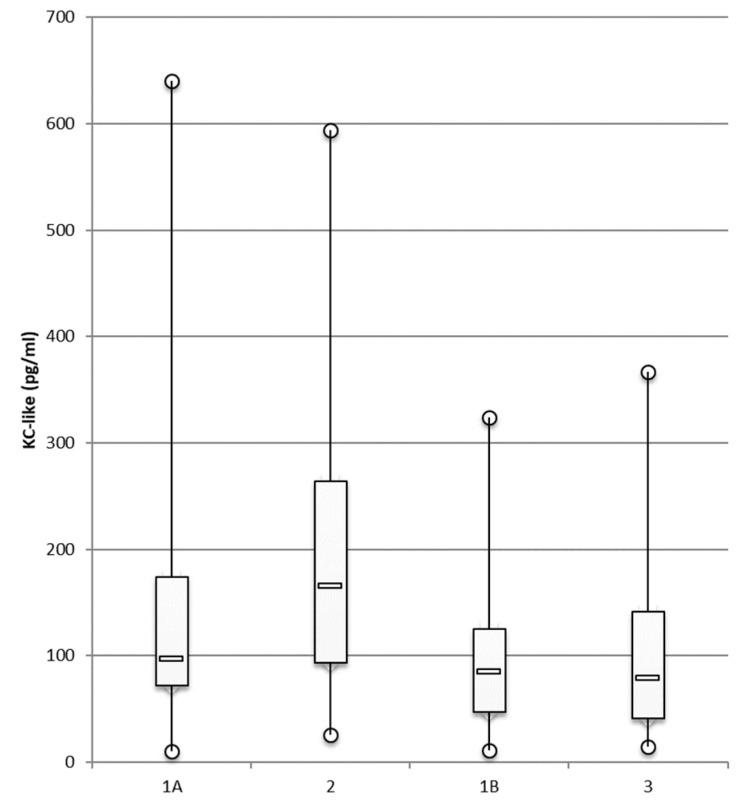
KC-like concentrations in ear canals of dogs suffering from atopic dermatitis/otitis and otodectic mange compared with those in healthy controls. Luminex^®^ technology was used to measure the concentrations of KC-like in the ear canals of healthy dogs, group 1A (*n* = 19 ears), and group 1B (*n* = 20 ears), and the concentrations were compared, respectively, with those in atopic dogs with otitis, group 2 (*n* = 14 ears), and in dogs with otodectic mange, group 3 (*n* = 20 ears) using the multiplex cytokine panel. Data are presented as box and whiskers (median, interquartile range, and min–max values).

Concentrations of KC-like (Figure 3), MCP-1, and IFN-γ (data not shown) did not differ significantly between atopic and healthy dogs.

### 3.4. Correlations between Clinical Scores (OTIS3, CADESI-04) and IL-8 Concentrations in Dogs with Atopic Otitis

A weak but positive correlation was found between IL-8 concentrations and the total OTIS3 score (r = 0.09, *p* = 0.33) and with erythema (r = 0.015, *p* = 0.69). A stronger significant positive correlation was found between IL-8 concentrations and the hyperplasia score (r = 0.39, *p* = 0.025).

No positive correlation was found between the CADESI-04 score and IL-8 concentrations.

## 4. Discussion

Luminex^®^ assays are an innovative technique to easily assess the relative concentrations of soluble mediators such as cytokines. Luminex^®^ allows rapid identification, requires significantly smaller sample volumes, and assay times are shorter, thus enabling higher throughput. Moreover, for cytokine profiling, the results are as accurate as those of an ELISA and more reproducible than a microarray [22]. Cytokine analysis has contributed significantly to our understanding of the pathophysiology of immune or non-immune diseases in both human and veterinary medicine. Regarding canine AD, the identification of cytokines is promising both in terms of diagnostic applications [23] and therapeutic targets [24].

In this study, among the 13 cytokines measured by multiplex assay, IFN-gamma, KC-like, and MCP-1 were identified at low levels and no significant difference was observed between the groups. However, IL-8 and IL-10 concentrations were significantly higher in the ear canals of dogs suffering from atopic otitis than in healthy dogs. Of these two cytokines, IL-8 was considered more relevant because it was identified at higher concentrations. Furthermore, overexpression of IL-8 was confirmed by an ELISA. The complementary ELISA method was used to confirm the results obtained with Luminex^®^, suggesting it is an accessible method for the routine dosage of IL-8 in the ear canals of atopic dogs.

Irrespective of the method, IL-8 expression was significantly higher in the ear canals of atopic dogs than in healthy dogs. IL-8 concentrations were also significantly correlated with the hyperplasia parameter, a well-established clinical marker of inflammation in canine otitis externa; this result highlights the significance of hyperplasia when performing the aural examination. In the literature, increased concentrations of cutaneous IL-8 have already been identified in human inflammatory skin diseases, particularly in human atopic dermatitis [25]. In the same study, IL-8 levels decreased after the administration of corticosteroids. Interestingly, concentrations of IL-8 have also been found in serum of atopic children and the concentrations were correlated with disease severity [26]. Another investigation found overexpression of this chemokine in the stratum corneum of atopic patients, where the concentrations were also correlated with disease severity [27]. In veterinary medicine, Santoro et al. demonstrated increased production of IL-8 by keratinocytes in dogs following exposure to house dust mites [28]. In the same way, in atopic dogs, IL-8 transcription was enhanced in a canine keratinocyte cell line after stimulation with Der f 1, a major allergen of *Dermatophagoides farinae* [29]. In a recent pilot study, concentrations of cytokines were measured by a Luminex assay in the tears and the conjunctiva of atopic dogs and compared with the concentrations detected in normal dogs. IL-8 was found to be over-expressed in conjunctiva and tears of atopic dogs despite subtle conjunctival symptoms [30]. Finally, a recent study showed that the plasma concentrations of IL-8, IL-7, and IL-15 cytokines were elevated in canine AD patients compared to in control dogs [31]. In our study, the correlation between IL-8 content and otitis severity (OTIS3 score) was weak, possibly due to the low number of dogs. However, when considering more specifically inflammation, of which a gross expression is glandular hyperplasia, the correlation was higher (r = 0.39; *p* < 0.025).

However, these cohorts did not include any other inflammatory skin disease, which would have served as a positive control group. Indeed, IL-8 is not specifically associated with atopic dermatitis; its main role is attracting neutrophils, and as such, it participates in general inflammatory conditions [32,33]. This fact is supported by the results obtained from the positive control group in the present study; chemokine IL-8 was overexpressed in the ear canals of dogs suffering from infestations of ear mites, and this differed significantly from healthy controls. Consequently, expression does not support a specific role of IL-8 in canine atopic otitis.

Enhanced expression of IL-10 in ear canals of atopic dogs was observed compared to in healthy controls. The difference was significant, but the levels were very low in both groups. These results are in accordance with high levels of IL-10 found in lesional atopic skin compared with non-lesional atopic skin [13]. However, reports in the literature regarding the role of IL-10 in AD are conflicting. Indeed, IL-10 can apparently promote either Th2-type inflammation or act as a regulatory cytokine and downregulate immune responses [13,34]. In ear canals, our results suggest a proinflammatory role, as concentrations were significantly higher in group 2 than in healthy dogs. Another hypothesis is that IL-10 is produced by keratinocytes to downregulate immune response, but in our study, the levels were too low to counteract the Th2 response.

A major limitation of our study is the small number of enrolled dogs. However, the aim of this pilot study was to demonstrate the detection ability of cytokines and chemokines in the ear canals of dogs suffering from otitis externa compared to healthy dogs, and to compare this expression according to the type of inflammation, i.e., allergic or not. Further investigations are now needed using larger numbers of dogs to confirm these data, to possibly detect other inflammatory biomarkers in atopic ear canals, for instance, IL-31 and cytokine implicated in pruritic skin conditions in humans and dogs [35]. Finally, researchers need to investigate the impact of topical treatments on cytokine expression and assess the efficacy of a topical and systemic treatments.

In this study, we intentionally selected only non-infected otitis in order to avoid the production of pro-inflammatory cytokines related to bacterial contamination/proliferation. Indeed, numerous studies have demonstrated the overexpression of pro-inflammatory cytokines by keratinocyte cell lines upon exposure to *Staphylococcus pseudintermedius* [36] and *S. aureus* in humans [37]. Evaluating cytokine expression during infected otitis would also be interesting.

## 5. Conclusions

In this study, the presence of inflammatory biomarkers was demonstrated in atopic otitis in dogs, especially the chemokine IL-8. Unlike on skin, the presence of inflammatory biomarkers has never previously been identified in the ear canals of atopic dogs. Overexpression of IL-8 was correlated with the severity of otitis, particularly with the hyperplasia score. In addition, we demonstrated overexpression of this chemokine in parasitic otitis in dogs.

This study is a proof of concept; further investigations with a larger number of dogs are now required to confirm these results and possibly to find other biomarkers involved in the pathogenesis of canine atopic otitis. Such inflammation biomarkers could help monitor otitis following topical treatment and even be therapeutic targets to prevent otitis in dogs suffering from atopic dermatitis.

## Figures and Tables

**Table 1:** Clinical characteristic of subjects. Data are presented as medians (interquartile range).

	Group 1. Healthy Dogs		Group 2. Atopic Otitis 8 Dogs (*n* = 14 Ears)	Group 3. *Otodectes* Otitis 12 Dogs (*n* = 20 Ears)
	Group 1A. 10 Dogs (*n* = 19 Ears)	Group 1B. 11 Dogs (*n* = 20 Ears)		
Age (years)	5.75 (3.5–8.5)	5 (2.0–6.0)	4.25 (2.65–5.25)	4 (2.9–5.0)
Gender	6 females (3 spayed), 4 neutered males	5 females (2 spayed), 6 entire males	1 spayed female, 7 entire males	4 entire females, 8 entire males
Weight (kg)	19.4 (11.6–25.8)	24 (20.0–29.0)	16.8 (12.1–29.1)	15.2 (13.6–17.3)
Breeds	1 Fox terrier, 1 Berger Blanc Suisse, 3 crossbreeds, 1 Cairn terrier, 1 American Staffordshire terrier, 1 Border terrier, 2 Border Collies	2 Griffons, 4 Bleus de Gascogne, 1 Bruno du Jura, 1 Golden retriever, 1 Saint Bernard, 1 Bichon Maltese, 1 crossed Beauceron	1 Beagle, 1 Labrador, 2 English Cocker spaniels, 1 Jack Russel terrier, 1 French Bulldog, 1 German Shepherd, 1 Golden retriever	11 Beagles, 1 Jack Russel terrier
CADESI-04	2 (1–3)	5 (4.5–6.5)	24 (16–36)	3.5 (2.8–6)
OTIS3	0 (0–1)	1 (0.25–1)	4 (3–4)	1.5 (1–2)
VAS score	0 (0–0)	0 (0–0)	2.9 (1.5–3.8)	0.8 (0.7–1.2)

CADESI, Canine Atopic Dermatitis Extent and Severity Index; OTIS3, Otitis Index Score; VAS, Visual Analogue Scale.

## Data Availability

The data presented in this study are available from the corresponding author upon request. The data are not publicly available due to the need to maintain patient confidentiality.
